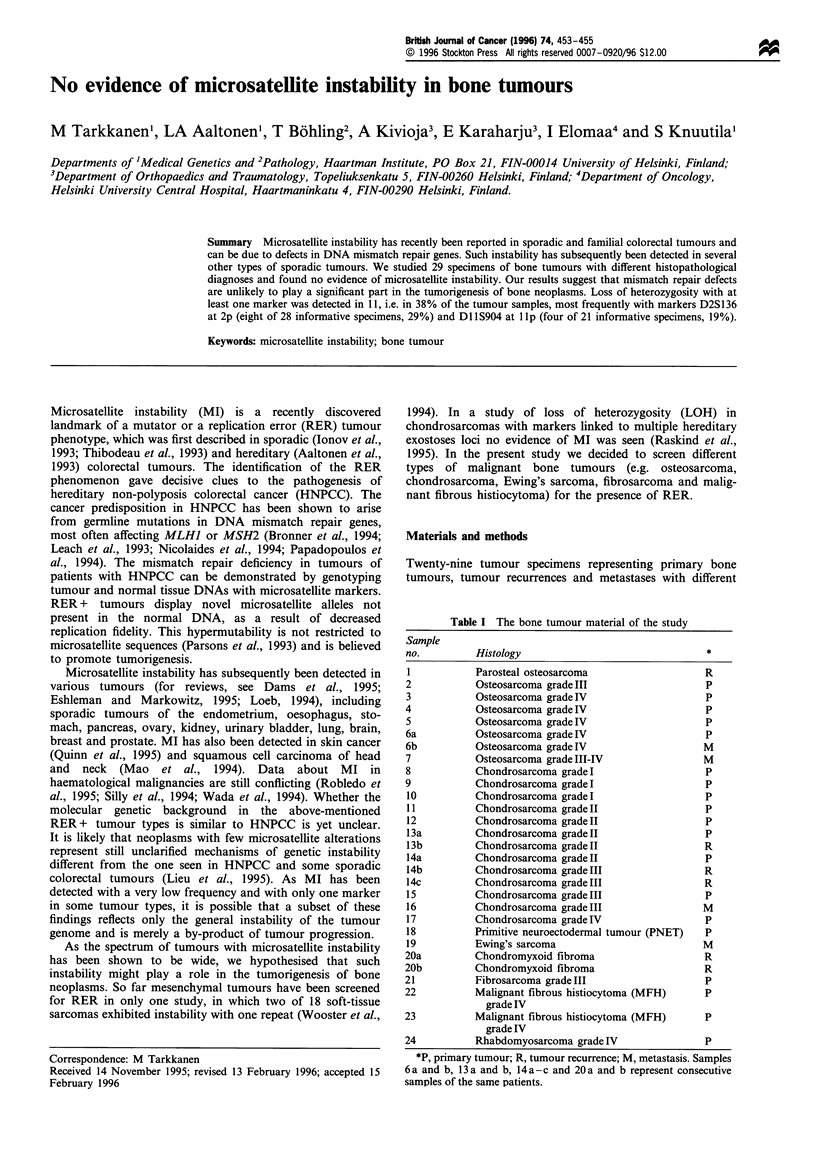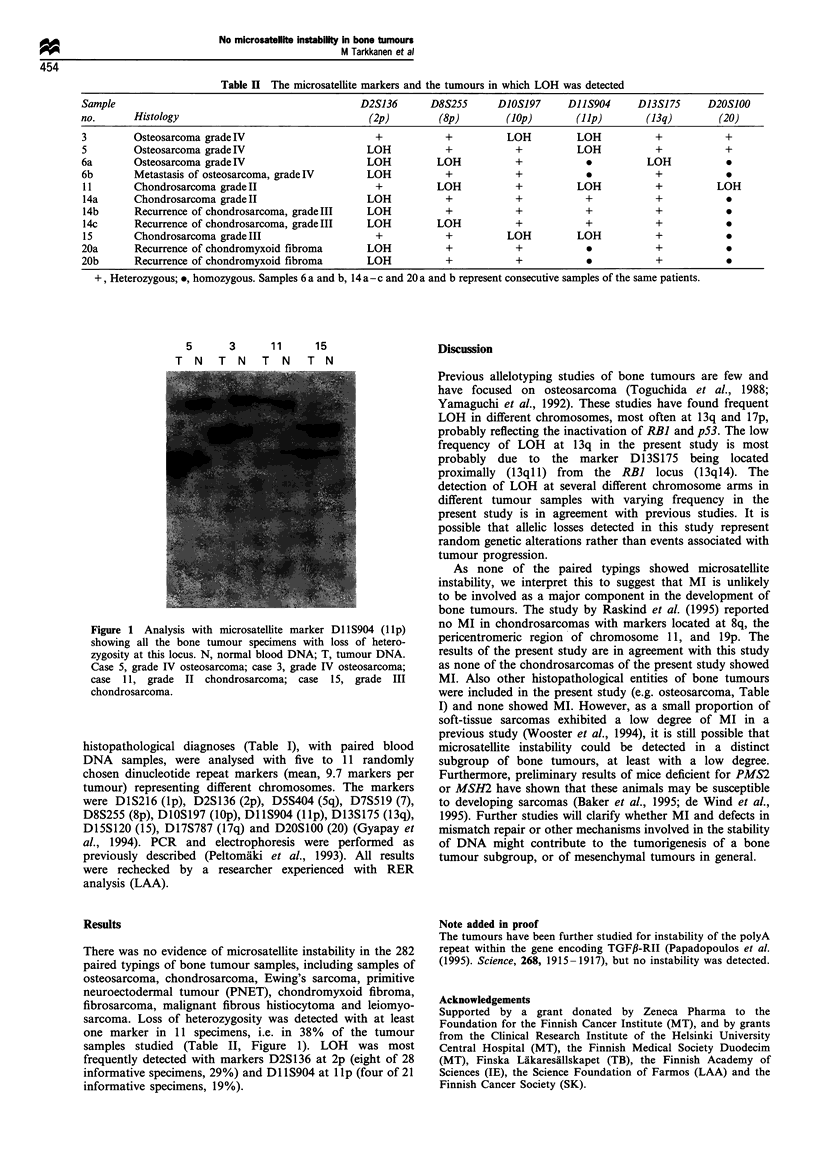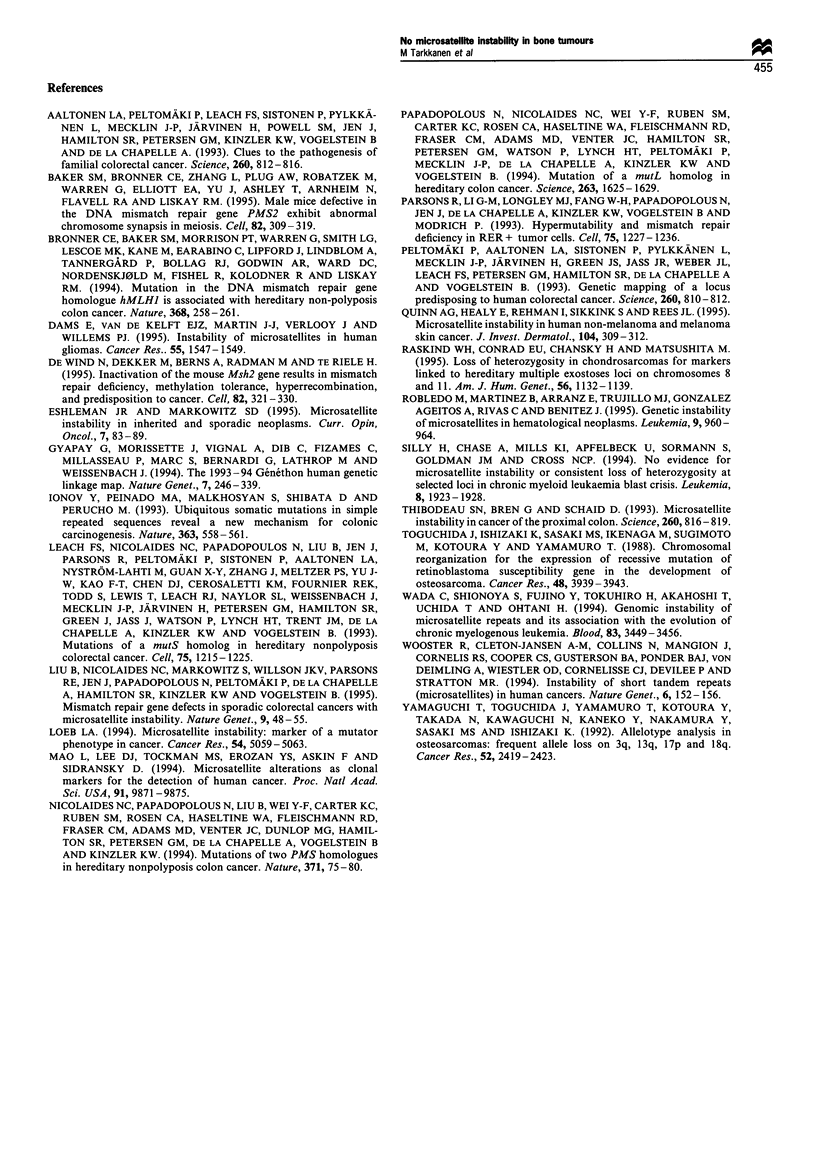# No evidence of microsatellite instability in bone tumours.

**DOI:** 10.1038/bjc.1996.380

**Published:** 1996-08

**Authors:** M. Tarkkanen, L. A. Aaltonen, T. Böhling, A. Kivioja, E. Karaharju, I. Elomaa, S. Knuutila

**Affiliations:** Department of Medical Genetics, Haartman Institute, University of Helsinki, Finland.

## Abstract

**Images:**


British Journal of Cancer (1996) 74, 453-455
© 1996 Stockton Press All rights reserved 0007-0920/96 $12.00

### No evidence of microsatellite instability in bone tumours

M Tarkkanen[1], LA Aaltonen[1], T Böhling[2], A Kivioja[3], E Karaharju[3], I Elomaa[4] and S Knuutila[1]

*Departments of [1]Medical Genetics and [2]Pathology, Haartman Institute, PO Box 21, FIN-00014 University of Helsinki, Finland; [3]Department of Orthopaedics and Traumatology, Topeliuksenkatu 5, FIN-00260 Helsinki, Finland; [4]Department of Oncology, Helsinki University Central Hospital, Haartmaninkatu 4, FIN-00290 Helsinki, Finland.*

**Summary** Microsatellite instability has recently been reported in sporadic and familial colorectal tumours and can be due to defects in DNA mismatch repair genes. Such instability has subsequently been detected in several other types of sporadic tumours. We studied 29 specimens of bone tumours with different histopathological diagnoses and found no evidence of microsatellite instability. Our results suggest that mismatch repair defects are unlikely to play a significant part in the tumorigenesis of bone neoplasms. Loss of heterozygosity with at least one marker was detected in 11, i.e. in 38% of the tumour samples, most frequently with markers D2S136 at 2p (eight of 28 informative specimens, 29%) and D11S904 at 11p (four of 21 informative specimens, 19%).

**Keywords:** microsatellite instability; bone tumour

Microsatellite instability (MI) is a recently discovered landmark of a mutator or a replication error (RER) tumour phenotype, which was first described in sporadic (Ionov *et al.*, 1993; Thibodeau *et al.*, 1993) and hereditary (Aaltonen *et al.*, 1993) colorectal tumours. The identification of the RER phenomenon gave decisive clues to the pathogenesis of hereditary non-polyposis colorectal cancer (HNPCC). The cancer predisposition in HNPCC has been shown to arise from germline mutations in DNA mismatch repair genes, most often affecting *MLH1* or *MSH2* (Bronner *et al.*, 1994; Leach *et al.*, 1993; Nicolaides *et al.*, 1994; Papadopoulos *et al.*, 1994). The mismatch repair deficiency in tumours of patients with HNPCC can be demonstrated by genotyping tumour and normal tissue DNAs with microsatellite markers. RER+ tumours display novel microsatellite alleles not present in the normal DNA, as a result of decreased replication fidelity. This hypermutability is not restricted to microsatellite sequences (Parsons *et al.*, 1993) and is believed to promote tumorigenesis.

Microsatellite instability has subsequently been detected in various tumours (for reviews, see Dams *et al.*, 1995; Eshleman and Markowitz, 1995; Loeb, 1994), including sporadic tumours of the endometrium, oesophagus, stomach, pancreas, ovary, kidney, urinary bladder, lung, brain, breast and prostate. MI has also been detected in skin cancer (Quinn *et al.*, 1995) and squamous cell carcinoma of head and neck (Mao *et al.*, 1994). Data about MI in haematological malignancies are still conflicting (Robledo *et al.*, 1995; Silly *et al.*, 1994; Wada *et al.*, 1994). Whether the molecular genetic background in the above-mentioned RER+ tumour types is similar to HNPCC is yet unclear. It is likely that neoplasms with few microsatellite alterations represent still unclarified mechanisms of genetic instability different from the one seen in HNPCC and some sporadic colorectal tumours (Lieu *et al.*, 1995). As MI has been detected with a very low frequency and with only one marker in some tumour types, it is possible that a subset of these findings reflects only the general instability of the tumour genome and is merely a by-product of tumour progression.

As the spectrum of tumours with microsatellite instability has been shown to be wide, we hypothesised that such instability might play a role in the tumorigenesis of bone neoplasms. So far mesenchymal tumours have been screened for RER in only one study, in which two of 18 soft-tissue sarcomas exhibited instability with one repeat (Wooster *et al.*, 1994). In a study of loss of heterozygosity (LOH) in chondrosarcomas with markers linked to multiple hereditary exostoses loci no evidence of MI was seen (Raskind *et al.*, 1995). In the present study we decided to screen different types of malignant bone tumours (e.g. osteosarcoma, chondrosarcoma, Ewing's sarcoma, fibrosarcoma and malignant fibrous histiocytoma) for the presence of RER.

### Materials and methods

Twenty-nine tumour specimens representing primary bone tumours, tumour recurrences and metastases with different

**Table I** The bone tumour material of the study

| *Sample no.* | *Histology* | * |
|---|---|---|
| 1 | Parosteal osteosarcoma | R |
| 2 | Osteosarcoma grade III | P |
| 3 | Osteosarcoma grade IV | P |
| 4 | Osteosarcoma grade IV | P |
| 5 | Osteosarcoma grade IV | P |
| 6a | Osteosarcoma grade IV | P |
| 6b | Osteosarcoma grade IV | M |
| 7 | Osteosarcoma grade III-IV | M |
| 8 | Chondrosarcoma grade I | P |
| 9 | Chondrosarcoma grade I | P |
| 10 | Chondrosarcoma grade I | P |
| 11 | Chondrosarcoma grade II | P |
| 12 | Chondrosarcoma grade II | P |
| 13a | Chondrosarcoma grade II | P |
| 13b | Chondrosarcoma grade II | R |
| 14a | Chondrosarcoma grade II | P |
| 14b | Chondrosarcoma grade III | R |
| 14c | Chondrosarcoma grade III | R |
| 15 | Chondrosarcoma grade III | P |
| 16 | Chondrosarcoma grade III | M |
| 17 | Chondrosarcoma grade IV | P |
| 18 | Primitive neuroectodermal tumour (PNET) | P |
| 19 | Ewing's sarcoma | M |
| 20a | Chondromyxoid fibroma | R |
| 20b | Chondromyxoid fibroma | R |
| 21 | Fibrosarcoma grade III | P |
| 22 | Malignant fibrous histiocytoma (MFH) grade IV | P |
| 23 | Malignant fibrous histiocytoma (MFH) grade IV | P |
| 24 | Rhabdomyosarcoma grade IV | P |

*P, primary tumour; R, tumour recurrence; M, metastasis. Samples 6a and b, 13a and b, 14a–c and 20a and b represent consecutive samples of the same patients.

Correspondence: M Tarkkanen
Received 14 November 1995; revised 13 February 1996; accepted 15 February 1996

**Table II** The microsatellite markers and the tumours in which LOH was detected

| *Sample no.* | *Histology* | *D2S136 (2p)* | *D8S255 (8p)* | *D10S197 (10p)* | *D11S904 (11p)* | *D13S175 (13q)* | *D20S100 (20)* |
|---|---|---|---|---|---|---|---|
| 3 | Osteosarcoma grade IV | + | + | LOH | LOH | + | + |
| 5 | Osteosarcoma grade IV | LOH | + | + | LOH | + | + |
| 6a | Osteosarcoma grade IV | LOH | LOH | + | • | LOH | • |
| 6b | Metastasis of osteosarcoma, grade IV | LOH | + | + | • | + | • |
| 11 | Chondrosarcoma grade II | + | LOH | + | LOH | + | LOH |
| 14a | Chondrosarcoma grade II | LOH | + | + | + | + | • |
| 14b | Recurrence of chondrosarcoma, grade III | LOH | + | + | + | + | • |
| 14c | Recurrence of chondrosarcoma, grade III | LOH | LOH | + | + | + | • |
| 15 | Chondrosarcoma grade III | + | + | LOH | LOH | + | • |
| 20a | Recurrence of chondromyxoid fibroma | LOH | + | + | • | + | • |
| 20b | Recurrence of chondromyxoid fibroma | LOH | + | + | • | + | • |

+, Heterozygous; •, homozygous. Samples 6 a and b, 14 a–c and 20 a and b represent consecutive samples of the same patients.

**Figure 1** Analysis with microsatellite marker D11S904 (11p) showing all the bone tumour specimens with loss of heterozygosity at this locus. N, normal blood DNA; T, tumour DNA. Case 5, grade IV osteosarcoma; case 3, grade IV osteosarcoma; case 11, grade II chondrosarcoma; case 15, grade III chondrosarcoma.

histopathological diagnoses (Table I), with paired blood DNA samples, were analysed with five to 11 randomly chosen dinucleotide repeat markers (mean, 9.7 markers per tumour) representing different chromosomes. The markers were D1S216 (1p), D2S136 (2p), D5S404 (5q), D7S519 (7), D8S255 (8p), D10S197 (10p), D11S904 (11p), D13S175 (13q), D15S120 (15), D17S787 (17q) and D20S100 (20) (Gyapay *et al.*, 1994). PCR and electrophoresis were performed as previously described (Peltomäki *et al.*, 1993). All results were rechecked by a researcher experienced with RER analysis (LAA).

### Results

There was no evidence of microsatellite instability in the 282 paired typings of bone tumour samples, including samples of osteosarcoma, chondrosarcoma, Ewing's sarcoma, primitive neuroectodermal tumour (PNET), chondromyxoid fibroma, fibrosarcoma, malignant fibrous histiocytoma and leiomyosarcoma. Loss of heterozygosity was detected with at least one marker in 11 specimens, i.e. in 38% of the tumour samples studied (Table II, Figure 1). LOH was most frequently detected with markers D2S136 at 2p (eight of 28 informative specimens, 29%) and D11S904 at 11p (four of 21 informative specimens, 19%).

### Discussion

Previous allelotyping studies of bone tumours are few and have focused on osteosarcoma (Toguchida *et al.*, 1988; Yamaguchi *et al.*, 1992). These studies have found frequent LOH in different chromosomes, most often at 13q and 17p, probably reflecting the inactivation of *RB1* and *p53*. The low frequency of LOH at 13q in the present study is most probably due to the marker D13S175 being located proximally (13q11) from the *RB1* locus (13q14). The detection of LOH at several different chromosome arms in different tumour samples with varying frequency in the present study is in agreement with previous studies. It is possible that allelic losses detected in this study represent random genetic alterations rather than events associated with tumour progression.

As none of the paired typings showed microsatellite instability, we interpret this to suggest that MI is unlikely to be involved as a major component in the development of bone tumours. The study by Raskind *et al.* (1995) reported no MI in chondrosarcomas with markers located at 8q, the pericentromeric region of chromosome 11, and 19p. The results of the present study are in agreement with this study as none of the chondrosarcomas of the present study showed MI. Also other histopathological entities of bone tumours were included in the present study (e.g. osteosarcoma, Table I) and none showed MI. However, as a small proportion of soft-tissue sarcomas exhibited a low degree of MI in a previous study (Wooster *et al.*, 1994), it is still possible that microsatellite instability could be detected in a distinct subgroup of bone tumours, at least with a low degree. Furthermore, preliminary results of mice deficient for *PMS2* or *MSH2* have shown that these animals may be susceptible to developing sarcomas (Baker *et al.*, 1995; de Wind *et al.*, 1995). Further studies will clarify whether MI and defects in mismatch repair or other mechanisms involved in the stability of DNA might contribute to the tumorigenesis of a bone tumour subgroup, or of mesenchymal tumours in general.

### Note added in proof

The tumours have been further studied for instability of the polyA repeat within the gene encoding TGF$\beta$-RII (Papadopoulos *et al.* (1995). *Science*, **268**, 1915–1917), but no instability was detected.

### Acknowledgements

Supported by a grant donated by Zeneca Pharma to the Foundation for the Finnish Cancer Institute (MT), and by grants from the Clinical Research Institute of the Helsinki University Central Hospital (MT), the Finnish Medical Society Duodecim (MT), Finska Läkaresällskapet (TB), the Finnish Academy of Sciences (IE), the Science Foundation of Farmos (LAA) and the Finnish Cancer Society (SK).

### References

AALTONEN LA, PELTOMÄKI P, LEACH FS, SISTONEN P, PYLKKÄNEN L, MECKLIN J-P, JÄRVINEN H, POWELL SM, JEN J, HAMILTON SR, PETERSEN GM, KINZLER KW, VOGELSTEIN B AND DE LA CHAPELLE A. (1993). Clues to the pathogenesis of familial colorectal cancer. *Science*, **260**, 812–816.

BAKER SM, BRONNER CE, ZHANG L, PLUG AW, ROBATZEK M, WARREN G, ELLIOTT EA, YU J, ASHLEY T, ARNHEIM N, FLAVELL RA AND LISKAY RM. (1995). Male mice defective in the DNA mismatch repair gene *PMS2* exhibit abnormal chromosome synapsis in meiosis. *Cell*, **82**, 309–319.

BRONNER CE, BAKER SM, MORRISON PT, WARREN G, SMITH LG, LESCOE MK, KANE M, EARABINO C, LIPFORD J, LINDBLOM A, TANNERGÅRD P, BOLLAG RJ, GODWIN AR, WARD DC, NORDENSKJØLD M, FISHEL R, KOLODNER R AND LISKAY RM. (1994). Mutation in the DNA mismatch repair gene homologue ***hMLH1*** is associated with hereditary non-polyposis colon cancer. *Nature*, **368**, 258–261.

DAMS E, VAN DE KELFT EJZ, MARTIN J-J, VERLOOY J AND WILLEMS PJ. (1995). Instability of microsatellites in human gliomas. *Cancer Res.*. **55**, 1547–1549.

DE WIND N, DEKKER M, BERNS A, RADMAN M AND TE RIELE H. (1995). Inactivation of the mouse *Msh2* gene results in mismatch repair deficiency, methylation tolerance, hyperrecombination, and predisposition to cancer. *Cell*, **82**, 321–330.

ESHLEMAN JR AND MARKOWITZ SD (1995). Microsatellite instability in inherited and sporadic neoplasms. *Curr. Opin, Oncol.*, **7**, 83–89.

GYAPAY G, MORISSETTE J, VIGNAL A, DIB C, FIZAMES C, MILLASSEAU P, MARC S, BERNARDI G, LATHROP M AND WEISSENBACH J. (1994). The 1993–94 Généthon human genetic linkage map. *Nature Genet.*, **7**, 246–339.

IONOV Y, PEINADO MA, MALKHOSYAN S, SHIBATA D AND PERUCHO M. (1993). Ubiquitous somatic mutations in simple repeated sequences reveal a new mechanism for colonic carcinogenesis. *Nature*, **363**, 558–561.

LEACH FS, NICOLAIDES NC, PAPADOPOULOS N, LIU B, JEN J, PARSONS R, PELTOMÄKI P, SISTONEN P, AALTONEN LA, NYSTRÖM-LAHTI M, GUAN X-Y, ZHANG J, MELTZER PS, YU J-W, KAO F-T, CHEN DJ, CEROSALETTI KM, FOURNIER REK, TODD S, LEWIS T, LEACH RJ, NAYLOR SL, WEISSENBACH J, MECKLIN J-P, JÄRVINEN H, PETERSEN GM, HAMILTON SR, GREEN J, JASS J, WATSON P, LYNCH HT, TRENT JM, DE LA CHAPELLE A, KINZLER KW AND VOGELSTEIN B. (1993). Mutations of a *mutS* homolog in hereditary nonpolyposis colorectal cancer. *Cell*, **75**, 1215–1225.

LIU B, NICOLAIDES NC, MARKOWITZ S, WILLSON JKV, PARSONS RE, JEN J, PAPADOPOLOUS N, PELTOMÄKI P, DE LA CHAPELLE A, HAMILTON SR, KINZLER KW AND VOGELSTEIN B. (1995). Mismatch repair gene defects in sporadic colorectal cancers with microsatellite instability. *Nature Genet.*, **9**, 48–55.

LOEB LA. (1994). Microsatellite instability: marker of a mutator phenotype in cancer. *Cancer Res.*, **54**, 5059–5063.

MAO L, LEE DJ, TOCKMAN MS, EROZAN YS, ASKIN F AND SIDRANSKY D. (1994). Microsatellite alterations as clonal markers for the detection of human cancer. *Proc. Natl Acad. Sci. USA*, **91**, 9871–9875.

NICOLAIDES NC, PAPADOPOLOUS N, LIU B, WEI Y-F, CARTER KC, RUBEN SM, ROSEN CA, HASELTINE WA, FLEISCHMANN RD, FRASER CM, ADAMS MD, VENTER JC, DUNLOP MG, HAMILTON SR, PETERSEN GM, DE LA CHAPELLE A, VOGELSTEIN B AND KINZLER KW. (1994). Mutations of two *PMS* homologues in hereditary nonpolyposis colon cancer. *Nature*, **371**, 75–80.

PAPADOPOLOUS N, NICOLAIDES NC, WEI Y-F, RUBEN SM, CARTER KC, ROSEN CA, HASELTINE WA, FLEISCHMANN RD, FRASER CM, ADAMS MD, VENTER JC, HAMILTON SR, PETERSEN GM, WATSON P, LYNCH HT, PELTOMÄKI P, MECKLIN J-P, DE LA CHAPELLE A, KINZLER KW AND VOGELSTEIN B. (1994). Mutation of a *mutL* homolog in hereditary colon cancer. *Science*, **263**, 1625–1629.

PARSONS R, LI G-M, LONGLEY MJ, FANG W-H, PAPADOPOLOUS N, JEN J, DE LA CHAPELLE A, KINZLER KW, VOGELSTEIN B AND MODRICH P. (1993). Hypermutability and mismatch repair deficiency in RER+ tumor cells. *Cell*, **75**, 1227–1236.

PELTOMÄKI P, AALTONEN LA, SISTONEN P, PYLKKÄNEN L, MECKLIN J-P, JÄRVINEN H, GREEN JS, JASS JR, WEBER JL, LEACH FS, PETERSEN GM, HAMILTON SR, DE LA CHAPELLE A AND VOGELSTEIN B. (1993). Genetic mapping of a locus predisposing to human colorectal cancer. *Science*, **260**, 810–812.

QUINN AG, HEALY E, REHMAN I, SIKKINK S AND REES JL. (1995). Microsatellite instability in human non-melanoma and melanoma skin cancer. *J. Invest. Dermatol.*, **104**, 309–312.

RASKIND WH, CONRAD EU, CHANSKY H AND MATSUSHITA M. (1995). Loss of heterozygosity in chondrosarcomas for markers linked to hereditary multiple exostoses loci on chromosomes 8 and 11. *Am. J. Hum. Genet.*, **56**, 1132–1139.

ROBLEDO M, MARTINEZ B, ARRANZ E, TRUJILLO MJ, GONZALEZ AGEITOS A, RIVAS C AND BENITEZ J. (1995). Genetic instability of microsatellites in hematological neoplasms. *Leukemia*, **9**, 960–964.

SILLY H, CHASE A, MILLS KI, APFELBECK U, SORMANN S, GOLDMAN JM AND CROSS NCP. (1994). No evidence for microsatellite instability or consistent loss of heterozygosity at selected loci in chronic myeloid leukaemia blast crisis. *Leukemia*, **8**, 1923–1928.

THIBODEAU SN, BREN G AND SCHAID D. (1993). Microsatellite instability in cancer of the proximal colon. *Science*, **260**, 816–819.

TOGUCHIDA J, ISHIZAKI K, SASAKI MS, IKENAGA M, SUGIMOTO M, KOTOURA Y AND YAMAMURO T. (1988). Chromosomal reorganization for the expression of recessive mutation of retinoblastoma susceptibility gene in the development of osteosarcoma. *Cancer Res.*, **48**, 3939–3943.

WADA C, SHIONOYA S, FUJINO Y, TOKUHIRO H, AKAHOSHI T, UCHIDA T AND OHTANI H. (1994). Genomic instability of microsatellite repeats and its association with the evolution of chronic myelogenous leukemia. *Blood*, **83**, 3449–3456.

WOOSTER R, CLETON-JANSEN A-M, COLLINS N, MANGION J, CORNELIS RS, COOPER CS, GUSTERSON BA, PONDER BAJ, VON DEIMLING A, WIESTLER OD, CORNELISSE CJ, DEVILEE P AND STRATTON MR. (1994). Instability of short tandem repeats (microsatellites) in human cancers. *Nature Genet.*, **6**, 152–156.

YAMAGUCHI T, TOGUCHIDA J, YAMAMURO T, KOTOURA Y, TAKADA N, KAWAGUCHI N, KANEKO Y, NAKAMURA Y, SASAKI MS AND ISHIZAKI K. (1992). Allelotype analysis in osteosarcomas: frequent allele loss on 3q, 13q, 17p and 18q. *Cancer Res.*, **52**, 2419–2423.